# The behavioral phenotype in a cohort of patients with chromosome 18 anomalies: a retrospective observational study

**DOI:** 10.1186/s13052-025-01902-2

**Published:** 2025-02-25

**Authors:** Beatrice Allegri, Paola Francesca Ajmone, Giovanni Michelini, Virginia Antonietti, Silvia Tornielli, Fabio Bruschi, Francesca Dall’Ara, Federico Monti, Donatella Milani, Paola Giovanna Vizziello, Maria Antonella Costantino

**Affiliations:** 1 Child and Adolescent Neuropsychiatry Service (UONPIA), Fondazione IRCCS Cà Granda Ospedale Maggiore Policlinico -SC, via Pace 9, Milan, 20122 Italy; 2 Child and Youth Lab, Sigmund Freud University of Milan, Via Ripa di Porta Ticinese 77, Milan, 20143 Italy; 3 Fondazione IRCCS Ca’ Granda Ospedale Maggiore Policlinico Milano - SC Pediatria Pneumoinfettivologia, via Commenda 9, Milan, 20122 Italy

**Keywords:** Behavioral phenotype, Chromosome 18, ID, Genotype–phenotype, Autism spectrum disorders (ASD), Neuropsychiatric assessment

## Abstract

**Background:**

Genetic syndromes resulting from chromosome 18 structural abnormalities constitute a broad spectrum of conditions characterized by significant clinical heterogeneity. Most studies in the literature focus on case reports and clinical observations; the present study aims to assess the cognitive, communicative, behavioral, and adaptive abilities of different chromosome 18 abnormalities. In addition, this work aims to identify phenotype-genotype correlations by comparing individuals with 18p deletion, 18q deletion, and 18p tetrasomy.

**Methods:**

The sample included 24 patients with a definite genetic diagnosis of 18p deletion (*N* = 6), 18q deletion (*N* = 9), or 18p tetrasomy (*N* = 8). The assessment is provided by using a specific protocol based on direct and indirect clinical assessment of patients. Differences in IQ/GQ indexes, adaptive behavior, CARS scores, and CBCL internalizing and externalizing symptoms were assessed using ANCOVAs with age as covariate.

**Results:**

Our results showed more significant cognitive and behavioral impairment in tetrasomy 18 than in the other two conditions. Conversely, in 18p deletion group, we found greater behaviorally susceptibility to develop autistic traits.

**Conclusion:**

These preliminary findings should raise clinicians’ awareness of the strengths and weaknesses of patients with chromosome 18 alterations, paving the way to targeted and more appropriate management.

**Supplementary Information:**

The online version contains supplementary material available at 10.1186/s13052-025-01902-2.

## Background

Genetic syndromes resulting from structural abnormalities of chromosome 18 constitute a broad spectrum of conditions characterized by facial dysmorphisms, developmental delay, intellectual disability, and behavioral problems. " The spectrum of structural abnormalities involving chromosome 18 includes 18p deletion, 18q deletion and 18 p tetrasomy. “.

This genetic variability produces phenotypic pictures characterized by significant clinical heterogeneity. Tetrasomy 18p (OMIM #614290) results from an abnormal extra chromosome composed of two copies of the short arm of chromosome 18, creating an isochromosome 18 present in each cell. The genetic condition is described in limited study participants or case reports and is estimated to occur in less than 1 in 625.000 [1–2]. Clinically, this condition is characterized mainly by developmental delay, microcephaly, abnormalities in muscle tone, feeding problems, genitourinary abnormalities, and dysmorphic features [3]. Genitourinary abnormalities account for 41% of all complications, including small kidneys, cryptorchidism, micropenis, and hypospadias [3] ). In contrast to the prevailing assumption that a severe cognitive impairment is a core feature of the condition [4–5–6]], some studies report that Intellectual Disability (ID) may range from mild to severe/profound. Regarding the behavioral phenotype, literature shows that difficulties in social and metacognitive developmental skills, and behavioral regulation problems, negatively affect these patients’ functioning [7]). Very little is known about the incidence of maladaptive behaviors in this population, and to date, the only published study is a clinical report by Swingle et al. [2006] [4] that included the presence of aggressive behavior and self-injury in the discussion of clinical presentation. In addition, most of the individuals with tetrasomy 18p show repetitive behaviors, communication and social interaction deficits similar to those found in autism spectrum disorders [7]).

The 18p deletion syndrome (OMIM #146390) is a rare chromosomal abnormality affecting about 1 in 56336 live births [1–2]( and has been well-described in literature with over 300 reported patients [8, 9](. The most common clinical features are cognitive impairment, speech delay, postnatal growth delay, dysmorphic features, brain and cardiac anomalies, and immunologic and endocrinological disorders [10]. Regarding neurocognitive characteristics, ID is the most frequently reported symptom, with Intelligence Quotient (IQ) that usually varies from borderline to severe cognitive impairment [11–12].

The 18q deletion syndrome (18q-, OMIM 601808) is a chromosomal disorder resulting from a segmental deletion on the long arm of chromosome 18 [13–14–15–16] ). Deletions can be classified into proximal interstitial deletion spanning the region between the centromere and the 46-Mb position (18q11.2‐18q21.1) and distal deletions spanning from the 46‐Mb position to qter (18q21.1‐qter) [17–18]). Distal deletions in turn can be distinguished into those including TCF4 gene and those which do not. Deletions including TCF4 gene are associated with a more severe clinical picture, characterized by a profound DD/ID and worse behavioral outcomes, when compared to patients affected by distal 18q- not including TCF4 [8]). These syndromes are characterized by ID, microcephaly, short stature, congenital aural atresia, foot deformities, hypotonia, and delayed myelination [19–20] ). These disorders are rare, with an estimated prevalence of 1:54764 [1–2](. Rojnueangnit et al. (2019) [21] reported as common presentations of 18q11‐q12 deletions developmental delay/intellectual disability (DD/ID) (82%), speech delay, autism spectrum disorders, attention deficits, and hyperactivity or other behavioral problems (30%); Specifically, N. Mahr (1996) [14] reported cognitive abilities ranging from borderline to severe ID with academic achievement similarly impaired. Performance in specific neuropsychological functions (including attention, novel problem solving, memory, language, visuomotor integration, and fine motor dexterity) is consistently in the moderately-to-severely impaired range. Challenging behaviours have been found to be common in both sexes, including aggressivity, hyperactivity, and temper tantrums. Daviss (2013) [22] reported that patients having terminal deletions of a small critical region of the long arm of chromosome 18 are highly likely to have mood disorders, anxiety, and to a lesser extent, externalizing disorders. The prevalence of autism in 18q- syndrome was found to be probably not greater than in other developmental disorders with a similar level of cognitive impairment. Moreover, Daviss et al. (2013) [22] hypothesized that patients with 18q deletions, intellectual impairments, and autistic symptoms might be relatively protected from developing a mood disorder [22]. Other studies confirm a higher incidence of several behavioral and psychiatric disorders, such as attention deficit hyperactivity disorder [23]. Finally, epilepsy is part of the clinical feature of patients with 18q deletion syndrome [24]). In most patients with 18q-deletion syndrome, seizures are focal, mainly occurring during the first years of life with a fair response to valproic acid or carbamazepine [25]).

Given these premises and from the studies reviewed in the literature, it is possible to highlight some common features in subjects carrying these genetic conditions. Most of the studies in the literature are based on clinical observations, and none were performed on a cohort of patients with different genotype using a “tailored” assessment protocol. Therefore, the present study aims to assess cognitive, communicative, behavioral, and adaptive skills of different abnormalities on chromosome 18 using a specific neuropsychiatric protocol based on direct and indirect clinical evaluation of the patients. The knowledge of the behavioral phenotype of the condition and its correlation with the genotype may allow an early, timely, and tailored treatment and intervention for these children and their families, with a preventive perspective improving their quality of life.

## Methods

### Participants

Twenty-four Italian patients (17 males and 7 females), with an age at evaluation ranging between 16 and 202 months (mean: 87.29 ± 61.6 with a median of 65 months), referred to our clinic for assessment and follow-up, were enrolled in the study. All patients had a diagnosis of deletion 18p, deletion 18q, or tetrasomy 18p as a result of genetic tests performed by FISH, Karyotype, or ARRAY method. In two patients, only a karyotype was performed, and in eight, a FISH analysis was performed, while arrayCGH was performed in 13 cases (assembly GRCh37). All patients were assessed at [Child and Adolescent Neuropsychiatric Service, Fondazione IRCCS Ca’ Granda Ospedale Maggiore Policlinico, Milan, Italy] **anonymized for blind revision**. The assessment protocol was administered to all subjects during outpatient visits by specialists skilled in complex disabilities and rare diseases. Their final inclusion in the study relied on the attainment of written informed consent, following a full explanation of the procedures undertaken. All patients were Caucasian from different Italian regions. Socioeconomic status was taken into account recording mother’s and father’s educational attainment and employment status as well as family marriage status. Approximately half of parents had a Bachelor or Master degree (50.0% of the mothers and 45.8% of the fathers); 66.7% of mothers and 83.3% of the fathers were employed and all parents were married at the time of their son’s evaluation.

Regarding instrumental assessment in our sample, only 11 out of 24 patients (45%) had an encephalic MRI, which revealed only nonspecific abnormalities, dysmorphisms of the corpus callosum, abnormalities of myelination, and dilatation of the ventricles. These data align with the literature (Prateek et al., 2024)(Linnankivi et al., 2003). EEG was performed in only 7 patients (29%) and only in one case significant epileptiform abnormalities were found specifically in the central temporal area (patient with epilepsy treated with Valproate and Lamotrigine). Whereas examinations such as visual and auditory evoked potentials were performed in only 4 patients and nothing pathological emerged from them. As for the growth parameters, we know that in the 18p tetrasomy group, 50% (*n* = 4) were born at term, while 37% (*n* = 3) were preterm, and of these, 1 presents a low birth weight (1890 kg). Only 18 Tetrasomy 25% (*n* = 2) patients presented microcephaly at birth (CC under the 3° percentile). In the 18q deletion group, we know that 66% (*n* = 6) were born at term (for three patients because data are not available); 6 (66%) patients presented low birth weight. Finally, in the 18p deletion group, one was late preterm, and all the patients presented average birth weight. Our sample presents a complex clinical picture of medical conditions from birth. However, we can report the following medical conditions frequently observed in our study: respiratory and cardiac complications, seizures, orthopedic and dermatological issues, visual and auditory problems, endocrinological disorders, and bone abnormalities. Related to malformations, these patients mainly present multiple congenital anomalies: facial malformations, upper and lower limb abnormalities, genitourinary malformations (especially cryptorchidism), cardiac malformations, and a condition of congenital hypotonia.

### Procedures

All patients were assessed with a protocol tailored to the syndrome phenotype, based on direct and indirect tools, aiming at evaluating Intellectual Quotient (IQ), General Quotient of Development (GQ), communicative skills, behavioral aspects, and adaptive behavior. We analyzed and described each developmental area considering the whole cohort of patients to obtain a detailed and in-depth description of the behavioral phenotype. Moreover, we focused on identifying phenotype-genotype correlations by comparing individuals with deletion 18p, deletion 18q, and 18p tetrasomy. This comparison was possible because the genotype-related groups at the time of the last follow-up were homogeneous in age and number (although the 18p deletion group is older, there was no statistical difference).

#### Cognitive and developmental assessment

Two different scales were used to assess Intellectual Quotient (IQ) and General Quotient of Development (GQ): the Leiter International Performance Scales Revised– Leiter-R [26], and the Griffiths’ Scale [27]. We used the Griffiths’ Scale [27] to evaluate the general developmental quotient (GQ) in patients from 0 to 8 years old. The GQ comprises six sub-quotients, one for each investigated area (locomotor, personal-social, language, eye, hand coordination performance, and practical reasoning). The GQ identifies how children perform across developmental areas. The Leiter International Performance Scales Revised– Leiter R (age range 2–21 years) [26] is a non-verbal cognitive test. It is useful because it has a short version that does not require high attention skills. Moreover, it does not require verbal communication abilities and can be administered when communication difficulties are present or to non-speaking children. We decided to assess IQ using the Leiter scale because it may better highlight the real cognitive abilities of patients with communication and attention disorders [28]. Although the two measures do not perfectly correspond, we considered both as indices of children’s development. Both tests yielded a standardized quotient with M = 100 and SD = 15. In literature, using a combined IQ/GQ index is quite common if participants are at significantly different stages concerning the level of functioning [29–30–31]. In addition, the Griffiths GQ appeared to be a good predictor of later IQ [32].

#### Adaptive behaviour assessment

The Vineland Adaptive Behaviour Scale (VABS), in its Italian adaptation and validation [33], was used to assess adaptive behaviour. The VABS is a semi-structured interview for caregivers and allows to assess global adaptive behavior skills (Adaptive Behaviour Composite) and ability in four specific domains (Communication, Daily Living Skills, Socialisation, and Motor Skills).

#### Communication and language evaluation

We used the Communication domains of the VABS scale (expressive and receptive language) to assess expressive and receptive skills. It provides a standardized language age-equivalent score.

#### Behavioural assessment and autism spectrum disorders

Behavioural characteristics of the participants were assessed using the Child Behaviour Checklist– CBCL [34], while ASD (autism spectrum disorders) symptomatology was evaluated with the Childhood Autism Rating Scale, second version (CARS 2) [35]. Child Behaviour Checklist (CBCL) [34] was used to assess children’s Behavioural characteristics. The CBCL is a 100-item questionnaire completed by parents reflecting their point of view of the child’s behaviour at the time of administration and for the preceding three months. It provides a child’s behaviour profile considering eight different subscales: withdrawn behaviour, somatic complaints, anxiety/depressed behaviour, opposite behaviour, aggressive behaviour, social problems, thought problems, and attention problems. Single sub-scales can also be scored in terms of two broad grouping of symptoms: internalizing (consisting of anxious/depressed, withdrawn, emotionally reactive, somatic complaints) and externalizing (consisting of attention problems, aggressive behaviour, rule-breaking). The Childhood Autism Rating Scale (CARS 2) [35] is a behavior rating scale used to screen for autism spectrum disorder in clinical and research studies. This scale investigates the presence of behavioural, cognitive, and communicative characteristics associated with autism and rates their severity. Fifteen areas are considered, and for each area, the child is rated on a scale from 1 (*normal behavior*) to 4 (*severely abnormal behaviour*). It provides a final score ranging from 15 to 60 corresponding to three severity ranges of autistic features (no autism, mild autism, and moderate autism) with different clinical cut-offs according to the subject’s age (whether younger than 13 or older).

### Statistical analysis

One-way ANOVA between groups was performed to evaluate age difference between the three syndromes. Although this analysis was not significant (see Results), given the strong effect size, age was used as covariate in subsequent analyses.

Fisher’s Exact Tests for count data were performed to evaluate the association between socioeconomic data and the three syndromes. They were preferred over chi square tests because not all the requirements regarding expected and observed frequencies were met.

One-way between ANCOVAs were used to evaluate differences in IQ/GQ indexes, VABS standardized scores, CARS scores, and CBCL internalizing, externalizing, and total scores between patients with 18p deletion, 18q deletion, or 18p tetrasomy using age as covariate. Post-hoc were performed on significant comparisons using [36] Holm’s (1979) adjustment for p values. Power analysis showed that with 7 participants per group to reach a significance of 0.05 with a power of 0.80 an effect size of *f*^2^ = 0.49 (equivalent to an *eta*^2^ = 0.33) was needed. This power analysis was based on the smallest group, due to the quasi-experimental design, we performed unbalanced ANOVAs consisting of 7 participants with a deletion 18p, 9 with a deletion 18q and 8 with a tetrasomy 18p. These conditions are rare, so it was not possible, at the time of writing, to collect a larger sample and the authors are aware that even medium effect size differences will result in non-significant statistical results.

Effect size were reported as Generalized Eta Squared (ges: Olejnik and Algina, 2003) [37].

A correlation between IQ/GQ indexes and CARS scores was performed to investigate a general relationship between developmental/cognitive levels and the characteristics associated with autism. A similar analysis was conducted using dichotomized variables of intellectual functioning (average or borderline functioning vs. mild, moderate, severe, or profound disability) and autistic traits: CARS scores in the non-autistic range were classified as “without autistic traits’’ while scores with mild, moderate and severe autism were transformed as “with autistic traits.” All data analyses were conducted using R 4.2.3 [38] and RStudio 2023.03.0.386 [39] with packages effsize [40], emmeans [41], janitor [42], knitr [43], rstatix [44] and tidyverse [45].

## Results

Firstly, a one-way between ANOVA was performed to exclude significant age differences between the three syndromes (18p: 117.29 ± 68.03 months; 18q: 93.7 ± 58.56 months; Tetrasomy 18p: 53.88 ± 48.60). No significant difference was found (F (2,21) = 2.283; *p* =.127; ges = 0.179) even if the effect size must be considered.

Fisher’s Exact Tests for count data were performed to evaluate the association between socioeconomic data and the three syndromes. Mother’s educational attainment and employment as well as father’s employment didn’t show a significant association with the syndrome. Family status was not analyzed because all parents were married at the time of the evaluation. A significant association (*p* =.019) between father qualification and syndrome was found.

### Cognitive and developmental assessment

Our sample presents an intellectual disability of varying severity according to ICD-10 (no ID, 12.5%; Borderline, 12.5%; Mild, 37.5%; Moderate, 29.2%; Severe, 4.2%).

**Table 1:** Intellectual disability (according to ICD-10) levels

Intellectual Disability	*n*	percent
*No Intellectual Disability*	*3*	*12.5%*
*Borderline Intellectual functioning*	*3*	*12.5%*
*Mild intellectual disability*	*9*	*37.5%*
*Moderate intellectual disability*	*7*	*29.2%*
*Severe intellectual disability*	*2*	*8.3%*

ID, Intellectual Disability

Regarding the cognitive assessment, ANOVA showed a significant difference between the three groups (𝐹 (2,19) = 5.301, 𝑝 = 0.015; ges = 0.358). Post-hoc tests showed a statistically significant difference in cognitive level in tetrasomy 18p when compared with both deletions (*p* =.025 vs. 18p; *p* =.025vs. 18q) (Descriptive statistics are shown in Table 2).

**Table 2:** _ marginal means and standard errors resulting from ANCOVAs

The present study aims to delineate the behavioral phenotype in different chromosome 18 abnormalities using a protocol tailored for the characteristics of these patients and including both direct and indirect tools for a comprehensive neuropsychiatric evaluation. In the study, we considered three different categories of Scale	18p deletion	18q deletion	18p tetrasomy	Effect	F	df	*p*	*p* <.05	ges
				Age	2.180	(1, 19)	0.156		0.103
IQ/GQ index	70.615 +/- 7.439	67.599 +/- 5.712	41.865 +/- 6.469	Genetic	5.301	(2, 19)	0.015	*	0.358
				Age	0.908	(1, 17)	0.354		0.051
CARS-2 score	28.428 +/- 2.519	24.661 +/- 2.31	27.786 +/- 2.786	Genetic	0.717	(2, 17)	0.502		0.078
				Age	0.002	(1, 15)	0.967		0.000
CBCL Total score	61.869 +/- 3.174	65.726 +/- 2.85	54.784 +/- 3.274	Genetic	3.074	(2, 15)	0.076		0.291
				Age	0.272	(1, 15)	0.610		0.018
CBCL internalizing factors	62.388 +/- 4.075	66.472 +/- 3.659	46.561 +/- 4.203	Genetic	6.398	(2, 15)	0.010	*	0.460
				Age	1.777	(1, 15)	0.202		0.106
CBCL externalizing factors	59.129 +/- 2.766	58.323 +/- 2.484	48.827 +/- 2.853	Genetic	3.874	(2, 15)	0.044	*	0.341
				Age	12.958	(1, 19)	0.002	*	0.405
VABS Composite score	58.262 +/- 6.508	59.36 +/- 5.224	37.398 +/- 5.777	Genetic	4.339	(2, 19)	0.028	*	0.314
				Age	8.928	(1, 19)	0.008	*	0.320
VABS Communication (domain)	55.078 +/- 6.019	60.171 +/- 4.831	43.124 +/- 5.343	Genetic	2.731	(2, 19)	0.091		0.223
				Age	1.449	(1, 19)	0.243		0.071
VABS Receptive (sub-domain)	8.633 +/- 1.144	9.243 +/- 0.919	6.501 +/- 1.016	Genetic	1.998	(2, 19)	0.163		0.174
				Age	0.088	(1, 19)	0.770		0.005
VABS Expressive (sub-domain)	4.429 +/- 1.095	6.747 +/- 0.879	4.713 +/- 0.972	Genetic	1.854	(2, 19)	0.184		0.163
				Age	2.983	(1, 11)	0.112		0.213
VABS Written (sub-domain)	6.338 +/- 0.967	8.684 +/- 0.814	1.174 +/- 1.247	Genetic	12.734	(2, 11)	0.001	*	0.698
				Age	25.391	(1, 19)	0.000	*	0.572
VABS Daily Living (domain)	57.874 +/- 4.752	68.966 +/- 3.814	45.008 +/- 4.218	Genetic	8.602	(2, 19)	0.002	*	0.475
				Age	0.325	(1, 19)	0.575		0.017
VABS Personal (sub-domain)	2.856 +/- 1.529	6.084 +/- 1.227	2.889 +/- 1.357	Genetic	2.070	(2, 19)	0.154		0.179
				Age	3.304	(1, 19)	0.085		0.148
VABS Domestic (sub-domain)	9.284 +/- 1.141	12.755 +/- 0.916	10.313 +/- 1.013	Genetic	3.297	(2, 19)	0.059		0.258
				Age	28.649	(1, 19)	0.000	*	0.601
VABS Community (sub-domain)	9.656 +/- 1.136	9.028 +/- 0.912	7.351 +/- 1.009	Genetic	1.209	(2, 19)	0.321		0.113
				Age	18.333	(1, 19)	0.000	*	0.491
VABS Socialisation (domain)	62.13 +/- 5.33	58.747 +/- 4.279	49.812 +/- 4.732	Genetic	1.566	(2, 19)	0.235		0.141
				Age	15.243	(1, 19)	0.001	*	0.445
VABS Interper. rel. (sub-domain)	7.662 +/- 0.976	8.255 +/- 0.784	5.841 +/- 0.867	Genetic	2.110	(2, 19)	0.149		0.182
				Age	0.670	(1, 19)	0.423		0.034
VABS Play and leisure time (sub-domain)	6.236 +/- 1.325	6.104 +/- 1.064	6.456 +/- 1.177	Genetic	0.024	(2, 19)	0.977		0.002
				Age	25.858	(1, 19)	0.000	*	0.576
VABS Social Rules (sub-domain)	8.182 +/- 1.069	9.522 +/- 0.858	8.652 +/- 0.949	Genetic	0.539	(2, 19)	0.592		0.054
				Age	21.126	(1, 10)	0.001	*	0.679
VABS Motor skills (domain)	72.792 +/- 4.399	61.188 +/- 3.458	52.614 +/- 3.26	Genetic	6.392	(2, 10)	0.016	*	0.561
				Age	0.566	(1, 10)	0.469		0.054
VABS Gross (sub-domain)	7.933 +/- 0.473	7.93 +/- 0.372	7.258 +/- 0.35	Genetic	0.966	(2, 10)	0.413		0.162
				Age	7.507	(1, 10)	0.021	*	0.429
VABS Fine (sub-domain)	9.685 +/- 1.333	9.32 +/- 1.048	7.224 +/- 0.988	Genetic	1.364	(2, 10)	0.299		0.214
IQ, Intellective Quotient; CBCL, Child Behaviour Checklist; VABS, Vineland Adaptive Behaviour Scale									

### Behavioural and emotional assessment

No difference was found in CARS scores (F (2,17) = 0.717, 𝑝= 0.502; ges = 0.078) (Descriptive statistics are shown in Table 2). Three participants were excluded from the analysis because they were too young to be evaluated with CARS. Even using a dichotomized variable for autistic traits (“with” or “without autistic traits”), there was no association with the genetic condition. Due to the small-sized sample, a Fisher’s exact test was performed, but it showed no significance (*p* =.502). The relationship between developmental/cognitive levels and autistic traits was assessed at first using the Pearson coefficient: results showed a small (*r* = -.32) but not significant ($\:{t}_{18}=-1.435;p=.168$) correlation. Similar results were found dichotomizing CARS score in “with” or “without autistic traits” and applying an independent samples t-test with IQ/GQ score as dependent variable ($\:{t}_{18}=0.57;p=.576;d=0.26$ resulting in “small” effect size) (with ASD traits mean IQ/GQ: 58.88 $\:\pm\:$ 27.01 vs. without ASD traits mean IQ/GQ: 64.17 $\:\pm\:$ 14.63). One-way ANOVAs between groups were used to evaluate differences in behaviour, specifically in CBCL internalizing, externalizing, and total score. Five participants had missing or invalid values: two patients were too young and out of the range test, while three did not complete the questionnaires. ANOVAs showed significant differences between genetic conditions for internalizing symptoms (F (2,15) = 6.398; *p* =.010, ges = 0.460), no significant differences were found in the total score (F(2, 15) =3.074; *p* =.076, ges = 0.291), and for externalizing symptoms (F(2, 15) = 3.874; *p* =.044 ges = 0.341). Post-hoc tests showed a statistically significant difference in internalizing symptoms in tetrasomy 18p when compared with both deletions (*p* =.004 vs. 18p; *p* =.009 vs. 18q) and a statistically significant difference in externalizing symptoms in tetrasomy 18p when compared with both deletions (*p* =.039 vs. 18p; *p* =.039 vs. 18q). Regarding maladaptive behaviors from a qualitative point of view, our sample appears as follows: 33% (*n*=3) of the 18q deletion group exhibit challenging behavior (self- and other-directed aggression, oppositionality, and low frustration tolerance), but none of them are receiving pharmacological therapy. 57% (*n*=4) of the 18p deletion patients display challenging behavior, including self-injurious behavior, psychomotor agitation, and psychotic episodes, and some of these (*n*=2) are comorbid with autism. In this group, three patients are on pharmacological therapy (1. risperidone, 2. quetiapine and chlorpromazine, 3. lamotrigine and valproate [comorbidity with epilepsy]). In the 18p tetrasomy group, where the age of participants is lower, self-directed aggressive behavior and restricted interests result in only one patient, but without drug treatment.

(Descriptive statistics for behavioural assessment are shown in Table 2).

### Adaptive behaviour assessment

Regarding adaptive behavior, ANCOVAs showed a significant difference between the three groups in the VABS composite score (F(2, 19) = 4.339, *p* =.028, ges = 0.314). The Vineland subscale of the Writing subscale can be administered from age 36 while the Motor Skills subscale can be assessed up to age 7; this is why we have some missing data. Post-hoc tests showed a statistically significant difference in tetrasomy 18p when compared with deletion 18q (*p* =.037). A significant difference was found also in the Written subdomain F(2, 11) = 12.734, *p* =.001, ges = 0.698), with post-hoc tests showing a statistically significant difference in tetrasomy 18p when compared with both 18q and 18p deletions (*p* =.015 vs. 18p; *p* =.001 vs. 18q) and in Daily Living subdomain (F(2, 19) = 8.602, *p* =.002, ges = 0.475), with post-hoc tests reporting a statistically significant difference in daily living skills in tetrasomy 18p when compared with deletion 18q (*p* =.002). Finally, ANCOVAs showed a significant difference between the three groups (F(2, 10) = 6.392, *p* =.016, ges = 0.561) in the Motor subdomain and post-hoc tests showed a statistically significant difference in tetrasomy 18p when compared with deletion 18p (*p* =.015).

(Descriptive statistics are presented in Table 2).

## Discussion

chromosome 18 alteration.

: tetrasomy 18p, 18p deletion, and 18q deletion. In agreement with the literature, our sample presents an intellectual disability of varying severity according to ICD-10 (no ID, 12.5%; Borderline, 12.5%; Mild, 37.5%; Moderate, 29.2%; Severe, 4.2%) (Table 1). IQ analysis shows that patients with 18p tetrasomy have statistically significantly lower scores than the other two conditions, which aligns with the existing literature [4–5–6]. Concerning the presence of autistic traits assessed by CARS, no significant difference emerges between the three genetic conditions. A closer analysis of the distribution of scores shows a higher concentration of autistic subjects in 18p deletion (57.1%), lthough not statistically significant. In addition, the 18p deletion showed higher, but not statistically significant, score averages than the other conditions (see Table 2). No significant correlation (*r*=-.32) and association were found in our sample between cognitive level and the presence of autism in any of the three genetic groups. In fact, tetrasomy 18p exhibits lower cognitive levels but not relevant autistic traits. Concerning the behavior phenotype assessed by CBCL, significant differences were found with regard to internalizing symptoms and total score. In particular, the 18p tetrasomy reported lower internalizing symptoms when compared to the 18p and 18q deletions. The investigation of adaptive development in the three conditions revealed a statistically significant difference in tetrasomy 18p when compared with both deletions: tetrasomy 18p showed worse abilities in daily living skills (statistically significant difference) compared with deletion 18q and worse abilities in the Motor subdomain compared with deletion 18p. No differences in any of the three categories concerning communication skills were found. Conversely, 18p deletion, from a qualitative point of view, seems to show a higher risk/frequency of autistic traits. Our study expands the knowledge of specific features in different chromosome anomalies by searching for genotype-behavioral phenotype correlation. This study presents some strengths: first, a tailored assessment protocol is used to evaluate neuropsychiatric functioning specific to the characteristics already described in the literature of subjects with a genetic abnormality of chromosome 18. Secondly, the same protocol was administered to the whole sample, which allowed us to compare data obtained within the three genetic categories. Moreover, this study tried to define a genotype-phenotype correlation in a cohort of patients with chromosome 18 alteration syndrome and better delineate some specific features for each genetic condition. For these reasons, our study represents a novelty compared with what is already present in the literature. In fact, there are primarily case-report studies or studies with a small sample of subjects, which consider only one genetic category.

Notwithstanding the innovative aspects of this study, some limitations must be acknowledged. Although the sample we considered allowed us to perform analyses, it is small due to the rarity of these genetic conditions. This may lead to underpowered analyses as only large differences in the three groups will result in statistically significant hypothesis tests. Also, although the age difference between the three conditions was not significantly different for the three conditions, the effect size must still be considered. In addition, despite the patients enrolled coming from all over the country, being our hospital a national referral center for complex disabilities, we performed a single-center study.

Moreover, due to the retrospective nature of our study and the limited sample size, coupled with the lack of some necessary data, we were unable to conduct a proper analysis of the association between socio-economic status (SES) and disease outcomes, making this a limit of our study. Being aware that higher SES might impact outcomes in these patients because of additional, private rehabilitation treatments or better understanding of the disease, we therefore highlight the need of a better families’ SES profiling and search for possible correlations in future studies.

This work could be a starting point for a future multicentric study involving more national referral centers.

## Conclusions

In conclusion, in this study, different functioning profiles emerged for the three conditions. A more significant cognitive impairment was found in tetrasomy 18p than in the other two conditions. On the other side, we found that 18p deletion, from a qualitative point of view, seems to show a higher risk/frequency of autistic traits. Given that our sample is small, future studies are needed to confirm these results with the same type of sample and longitudinal focus.

Monitoring developmental trajectories of these patients on a regular basis might consent clinicians to put in place all habilitative/rehabilitative therapies the child will need, in order to attain, keep, or improve skills and functioning for daily living. The preliminary results of our study may guide clinicians to recognise patients’ strengths and weaknesses in order to develop a tailored assessment and to provide effective clinical management, with anticipatory guidance and better rehabilitative priorities. As an example, patients affected by tetrasomy 18p might benefit from a intensive habilitation therapy and behavioral interventions, in consideration of their severe intellectual disability and maladaptive behaviors; patients affected by 18p deletion, who frequently present with autistic symptoms, might benefit from ad hoc rehabilitations interventions.In fact, although these conditions are all characterized by developmental delay, a careful neuropsychiatric assessment that considers the specific features of neurodevelopmental profile (cognitive and adaptive abilities, behavioural aspects) allows us to elucidate relationships between the different areas that can be exploited for intervention purposes. It’s important to consider that the diagnosis of a rare genetic condition demands life-long medical, multidisciplinary as well as social care, and it requires integrated interventions aimed at addressing different needs of the patient which are meant to evolve alongside the developmental stages. Awareness of the early dysfunctional patterns which might pave the way for later neuropsychiatric impairments is the first step for timely, tailored and prevention-oriented interventions. Developmental milestones reflect individual maturation and specific therapeutic windows, which might be taken into account because the timing of interventions is essential for outcomes maximization ( for example the literature shows as a timely communicative intervention is priority in order to prevent challenger behaviors).

## Electronic supplementary material

Below is the link to the electronic supplementary material.

Supplementary Material 1

Supplementary Material 2

## Data Availability

The datasets used and/or analysed during the current study are available from the corresponding author on reasonable request.

## Abbreviations

ID: Intellectual disability
IQ: Intelligent quotient
DD: Developmental Disability
GQ: general Quotient of Development
VABS: Vineland Adaptive behaviour scale
CBCL: Child behaviour Checklist
ASD: Autismo spectrum disorders
CARS 2: Child Autism Rating Scale
