# Supplementary material for: The behavioral phenotype in a cohort of patients with chromosome 18 anomalies: a retrospective observational study
Source: Ital J Pediatr. 2025 Feb 25;51:60. doi: 10.1186/s13052-025-01902-2 (PMC11863595; doi:10.1186/s13052-025-01902-2)
Supplement: Supplementary file 1 — Supplementary Material 1 [file 13052_2025_1902_MOESM1_ESM.docx]

Milan, 11.28.24

Dear Prof. Selicorni

**RE: Reply to Revisions requested**

**ID Manuscript:** ITJP-D-24-00636

**Title: The behavioral phenotype in a cohort of patients with chromosome 18 anomalies: a retrospective observational study**

**Authors:** Beatrice Allegri^1^, Paola Francesca Ajmone^1,^ Giovanni Michelini^2^, Virginia Antonietti^1^, Silvia Tornielli^1^, Fabio Bruschi^1^, Francesca Dall’Ara^1^, Federico Monti^1^, Donatella Milani^3^, Paola Giovanna Vizziello^1^, Maria Antonella Costantino^1^.

Thank you very much for the insightful review of our manuscript and for reconsidering it for publication. My co-authors and I are delighted to have the opportunity to submit a revised version of our manuscript, which we believe has been improved due to the valuable comments made by the Reviewers. We have detailed how our revisions address each of the issues that, according to the Reviewers, warrant further consideration in the following responses to the Reviewers.

All authors agree with the content of the revised manuscript. This paper has not been previously published or accepted for publication, nor is it under consideration at another journal. We thank the Editor and the Reviewers for this opportunity to revise our manuscript and look forward to hearing from you in due course.

Yours sincerely,

Paola Francesca Ajmone (Corresponding author)

Mail: paola.ajmone@policlinico.mi.it

**Reviewer's Comments:**

**Reviewer 1 :**

**Comments to the Author:**

**The authors present a relevant series of patients with rare conditions involving chromosome 18, describing in detail the cognitive and behavioral features**

R: Thank you very much for the insightful review of our manuscript. Below we answered the Reviewer’s comments providing detailed explanations of the revisions made.

1. **In the introduction the authors underline the presence of maladaptive behaviors associated with the three conditions. In the results they should better illustrate the characteristics of these maladaptive behaviors in the sample, highlighting any differences in the three groups. The authors should also specify in how many patients it was necessary to use a pharmacological treatment for maladaptive behaviors, specifying the type of drug and the efficacy.**

R: We thank the Reviewer for having pointed out this important aspect that probably we have neglected to better explain in our previous version of the manuscript. We have added a more detailed qualitative description of maladaptive behaviors in the three conditions in the result section:

“Regarding maladaptive behaviors from a qualitative point of view, our sample appears as follows: 33% (n=3) of the 18q deletion group exhibit challenging behavior (self- and other-directed aggression, oppositionality, and low frustration tolerance), but none of them are receiving pharmacological therapy. 57% (n=4) of the 18p deletion patients display challenging behavior, including self-injurious behavior, psychomotor agitation, and psychotic episodes, and some of these (n=2) are comorbid with autism. In this group, three patients are on pharmacological therapy (1. risperidone, 2. quetiapine and chlorpromazine, 3. lamotrigine and valproate [comorbidity with epilepsy]). In the 18p tetrasomy group, where the age of participants is lower, self-directed aggressive behavior and restricted interests result in only one patient, but without drug treatment.”

**2.    The authors do not describe the instrumental assessment of patients. Were brain MRI, EEG and visual and auditory evoked potentials performed? I think the authors should explain these data because structural alterations of the central nervous system, such as brain malformation, or functional changes, such as EEG anomalies or epilepsy, and defects in visual or auditory function could be related to the variability of the cognitive and behavioural phenotype.**

R: We thank the reviewer for the suggestion with respect to instrumental investigations. In the manuscript, we reported the data that was available to us. We recognize the importance of the relationship between instrumental investigations and behavioral and cognitive phenotypes. However, this should deserve attention with future scientific work that can better focus on this association with a larger population using a standardized assessment protocol. The reviewer can find in the manuscript the following information about the instrumental assessment performed in the Participants section:

“Regarding instrumental assessment in our sample, only 11 out of 24 patients (45%) had an encephalic MRI, which revealed only nonspecific abnormalities, dysmorphisms of the corpus callosum, abnormalities of myelination, and dilatation of the ventricles**.**

These results are in line with the literature (Prateek M, Branson H, Yoon G , Shroff M, et al. Imaging Findings and MRI Patterns in a Cohort of 18q Chromosomal Abnormalities. American Journal of Neuroradiology October 2024, 45 (10) 1578-1585; Available from: <https://doi.org/10.3174/ajnr.A8361>; [Linnankivi](https://pubmed.ncbi.nlm.nih.gov/?term=Linnankivi+TT&cauthor_id=14508777) T, [Autti](https://pubmed.ncbi.nlm.nih.gov/?term=Autti+TH&cauthor_id=14508777) T, [Pihko](https://pubmed.ncbi.nlm.nih.gov/?term=Pihko+SH&cauthor_id=14508777) H, [Somer](https://pubmed.ncbi.nlm.nih.gov/?term=Somer+MS&cauthor_id=14508777) M, et al. 18q-syndrome: brain MRI shows poor differentiation of gray and white matter on T2-weighted images.J Magn Reson Imaging. 2003 Oct;18(4):414-9. Available from: doi: 10.1002/jmri.10383).

EEG was performed in only 7 patients (29% of the sample) and only in one case significant epileptiform abnormalities were found specifically in the central temporal area (patient with epilepsy treated with Valproate and Lamotrigine). Whereas examinations such as visual and auditory evoked potentials were performed in only 4 patients and nothing pathological emerged from them.

**Reviewer 2:**

**The manuscript aims to define the behavioral phenotype of patients with chromosomal 18 anomalies (18p deletion, 18q deletion, and 18 tetrasomy) and to outline some genotype-phenotype correlations.Despite involving the same chromosome, the three syndromes differ significantly in clinical presentation. Therefore, comparing the three patient groups is challenging, and we would expect to see different neurobehavioral phenotypes.**

R: We would like to thank the Reviewer for all the inspiring hints and meticulous revisions provided, that we have carefully considered to fit our paper with the high-quality standards of ITJP. Below, we answered the Reviewer’s comments, providing detailed explanations of the revisions made.

**If available, for a more comprehensive evaluation of the phenotype, it would be valuable to include the full clinical features of the patients, such as growth parameters, the presence of major malformations, or medical complications. Specifically, if available, neuroimaging data from the patients could also provide useful insights.**

R: We thank the Reviewer for pointing out these data that we probably needed to specify more in our previous manuscript version. The Reviewer can find the added information in the Participants section:

"As for the growth parameters, we know that in the 18p tetrasomy group, 50% (n=4) were born at term, while 37% (n=3) were preterm, and of these, 1 presents a low birth weight (1890 kg). Only 18 Tetrasomy 25% (n=2) patients presented microcephaly at birth (CC under the 3° percentile). In the 18q deletion group, we know that 66% (n=6) were born at term (for three patients because data are not available); 6 (66%) patients presented low birth weight. Finally, in the 18p deletion group, one was late preterm, and all the patients presented average birth weight. Our sample presents a complex clinical picture of medical conditions from birth. However, we can report the following medical conditions frequently observed in our study: respiratory and cardiac complications, seizures, orthopedic and dermatological issues, visual and auditory problems, endocrinological disorders, and bone abnormalities. Related to malformations, these patients mainly present multiple congenital anomalies: facial malformations, upper and lower limb abnormalities, genitourinary malformations (especially cryptorchidism), cardiac malformations, and a condition of congenital hypotonia. According to neuroimaging data, only 46% (n= 11) did a brain MRI, and nonspecific anomalies emerged, including corpus callosum dysmorphisms, myelination abnormalities, and ventricular dilation.”

**In the Methods section, 2.1 Participants, please specify the period of case enrollment and the type of "array method" used for genomic diagnosis.**

R. We thank the reviewer for the suggestion. We have specified in Participants section the case enrollment and the array method with the following sentence:

“In two patients, only a karyotype was performed, and in eight, a FISH analysis was performed, while arrayCGH was performed in 13 cases (assembly GRCh37).

**Below are some specific suggestions**

**Page 1 Line 55: "a broad spectrum of conditions characterized by facial dysmorphisms and abnormalities," delete "and abnormalities"**

R. On the reviewer’s suggestion we have deleted the following words "and abnormalities" in the manuscript

**Page 1 Line 58: "The discovery of the molecular basis of these conditions has revealed the presence of clear heterogeneity depending on the type of chromosomal alteration, either at the short or long arm of chromosome 18 or deletion or tetrasomy." Replace the sentence with the following " The spectrum of structural abnormalities involving chromosome 18 includes 18p deletion, 18q deletion and 18 p tetrasomy. "**

R.We thank the reviewer for the suggestion and we the sentence in the text were modified

**Page 3 Line 2: "from borderline to severe levels" replace levels with cognitive impairment**

R. We have modified the word in the text

**Page 3 Line 22: "when compared to patients affected by distal 18- not including TCF4" replace 18- with 18q-**

R. Done

**Page 4 Line 25: "of patients with different genetic mutations using a "tailored" assessment protocol." replace genetic mutations with genotypes**

R. we have replaced as suggested

**Table 1: please provide a row for patients with severe ID (2 patients, 4,2%)**

R. We have added the missing row in the table 1

**Page 11 Line 34: "considered three different categories of genetic alterations: tetrasomy 18p, 18p deletion," replace genetic with chromosome 18**

R. We proceeded to make the suggested change in manuscript

**Page 12 Line 24: "In general, chromosomal number (amount of transcribed gene) seems to have a greater influence on phenotype than the loss of genetic material (long and short arm)." There is no evidence to support this assumption; please remove it.**

R. We have removed the sentence from the text

**Page 12 Line 29: "Our study expands the knowledge of specific features in different mutations by searching for genotype-behavioral phenotype correlation." replace mutations with chromosome anomalies**

R. We have replaced with the term suggested

**Page 13 Line 44: "A more significant cognitive impairment was found in tetrasomy than in the other" replace tetrasomy with tetrasomy 18p**

R. We have replaced the word with tetrasomy 18p
