# Supplementary material for: The behavioral phenotype in a cohort of patients with chromosome 18 anomalies: a retrospective observational study
Source: Ital J Pediatr. 2025 Feb 25;51:60. doi: 10.1186/s13052-025-01902-2 (PMC11863595; doi:10.1186/s13052-025-01902-2)
Supplement: Supplementary file 2 — Supplementary Material 2 [file 13052_2025_1902_MOESM2_ESM.docx]

*Editor :   Professor Giovanni Corsello*

*Italian Journal of Pediatrics*

Milan, 08/06/24

Dear Editor,

Please find attached our manuscrip*t “The behavioral phenotype in a cohort of patients with chromosome 18 anomalies: a retrospective observational study”*which we would like to submit for publication as a original research article in *Italian Journal of Pediatrics.*

Also on behalf of my co-authors, I hereby declare that this paper has not been published previously or submitted for publication elsewhere. Moreover, the text has been reviewed by a native English speaker and the authors state that there is no potential conflict of interest. All the authors approved the final version of the manuscript.

I hope that you will find the paper suitable for publication and look forward to hearing from you in due course.

Yours faithfully,

Paola Francesca Ajmone

Child and Adolescent Neuropsychiatric Service (UONPIA)

Fondazione IRCCS Ca' Granda Ospedale Maggiore Policlinico, Milan, Italy

Via Pace 9, 20122, Milan, Italy.

Tel: +39-02-55034400

Fax: +39-02-55034421

Mail: paola.ajmone@policlinico.mi.it
